# Mesoporous magnetic secondary nanostructures as versatile adsorbent for efficient scavenging of heavy metals

**DOI:** 10.1038/srep17072

**Published:** 2015-11-25

**Authors:** Kakoli Bhattacharya, Devaborniny Parasar, Bholanath Mondal, Pritam Deb

**Affiliations:** 1 Department of Physics, Tezpur University (Central University), Tezpur-784028, India; 2 Department of Central Scientific Services, Indian Association for the Cultivation of Science, Jadavpur, Kolkata 700032, India

## Abstract

Porous magnetic secondary nanostructures exhibit high surface area because of the presence of plentiful interparticle spaces or pores. Mesoporous Fe_3_O_4_ secondary nanostructures (MFSNs) have been studied here as versatile adsorbent for heavy metal scavenging. The porosity combined with magnetic functionality of the secondary nanostructures has facilitated efficient heavy metal (As, Cu and Cd) remediation from water solution within a short period of contact time. It is because of the larger surface area of MFSNs due to the porous network in addition to primary nanostructures which provides abundant adsorption sites facilitating high adsorption of the heavy metal ions. The brilliance of adsorption property of MFSNs has been realized through comprehensive adsorption studies and detailed kinetics. Due to their larger dimension, MFSNs help in overcoming the Brownian motion which facilitates easy separation of the metal ion sorbed secondary nanostructures and also do not get drained out during filtration, thus providing pure water.

Presence of heavy metal in water has become a major area of concern worldwide. Unlike organic pollutants, they do not degrade biologically. Thus, presence of heavy metals, even at trace level, is believed to be a risk to the ecological environment as well as to the human health. Huge amount of metal-contaminated water is daily released into the environment as a result of various industrial, agricultural and human activities^1^. Arsenic, cadmium, copper, mercury, nickel, chromium, zinc, etc are some of the heavy metals most commonly found in waste water. Because of their high solubility in the aquatic environment, these heavy metals can be easily absorbed by living organisms and thus can possibly get accumulated in their body^2^. Presence of these heavy metals leads to deteoriation of the aesthetic quality of potable water and leads to serious health effects such as reduced growth and development, cancer, organ damage, etc. Utmost removal of these heavy metals from the environment is thus a very important issue.

Numerous approaches have been employed for removal of heavy metals from aqueous solution. Some of these methods are precipitation^3^, flotation^4^, ion exchange^5^,^6^, electrolytic method^7^ and so forth. However, most of the methods are costly and create sludge disposal problem. Adsorption is one of the most effective techniques which can overcome these drawbacks^8^. The major advantages of this method over other conventional techniques include its simple operation, low cost, high efficiency, least chemical and biological sludge generation, regeneration of sorbent and possibility of metal recovery^9^,^10^,^11^. Some of the commonly used adsorbents are minerals^12^, industrial by-products^13^, agricultural products and by-products^14^ etc. In comparison to all these adsorbents, nanosized metal oxides exhibit remarkably enhanced sorption efficiency. This is partly because of their large surface area and high activities caused by the size-quantization effect^15^. During the last decade, a number of nanosized metal oxides have been used for heavy metal adsorption such as CeO_2_ nanoparticles^16^, silica hollow nanospheres^17^, Al_2_O_3_ and MgO nanoparticles^18^ etc. But these nanosized metal oxides have certain drawbacks such as inconvenience in separation from the wastewater^8^. However, application of magnetic metal oxide based nanomaterial is much more promising for extraction of heavy metals from the water after treatment. Furthermore, these nanomaterials get attracted to an external magnetic field but loses magnetism as the applied field is removed, favouring their fast and convenient removal after getting bound with the heavy metals. Iron oxide nanosystem is a potential candidate for this purpose. Its inherent affinity towards some of the heavy metals and enhanced adsorption efficiency facilitates its easy extraction from water. However, with decrease in size to nano dimension, the response of the iron oxide particles towards the external magnetic field decreases deplorably, which is not sufficient to overcome Brownian motion and as such no efficient magnetic separation takes place^9^,^19^. In addition, it is a bit complicated to utilize small nanoparticles for heavy metal scavenging as it might be difficult to separate them from the water after adsorption^9^. In order to embark upon this problem, one practical approach is to prepare magnetic hierarchical structures. These hierarchical structures exhibit high specific surface area because of the presence of plentiful interparticle spaces or pore. Besides, due to its large size and weaker Brownian motion these structures show adequate magnetic response^20^. Introduction of pores on the surface of the nanomaterials serves as an additional benefit which will further enhance the surface area creating more adsorption sites. These pores, in addition to the magnetic property of the hierarchical nanostructures will help in achieving higher efficiency in removal of heavy metals from water solution. Though the nanoparticles will have higher efficiency of adsorption but the heavy metal adsorbed nanoparticles will pass through the filtration unit because of its fine dimension. Secondary nanostructures with hierarchical architecture will prevent draining out of metal adsorbed grains through the filters during filtration. Therefore, a system needs to be developed with comparatively higher dimension yet retaining the superior functionality of nanosystem.

Herein, we report the development of a porous magnetic (Fe_3_O_4_) hierarchical system by a cost effective hydrothermal method^21^. This study deals with the feasibility of using porous magnetic hierarchical system as a versatile heavy metals scavenger. The heavy metals scavenged in this work, are arsenic (V), copper (II) and cadmium (II). Although, it is known that As (III) is the predominant form of arsenic in underground water conditions, which is more toxic and mobile than As(V). But, since As (V) species is more easily removed, hence in all the arsenic removal processes the trivalent species is converted to its pentavalent form prior to the separation. Hence, in this study As (V) has been taken as the model heavy metal for its removal via adsorption. The adsorption behaviour of the MFSNs has been studied under different conditions such as adsorbent dosage, adsorbate concentration and contact time.

## Results

### Microstructural and magnetic property study of MFSNs

The formation mechanism of MFSNs involves three major steps viz., nucleation, growth of nucleus and self-assembly. Here, ethylene glycol serves as a solvent as well as a reducing agent. When it comes in contact with FeCl_3_ it reduces the iron precursor partly to Fe^2+^, leading to the concomitant presence of Fe^2+^ and Fe^3+^. Addition of NaOAc creates an abundance of nucleation sites which in turn accelerates the nucleation process of the nanocrystals. Under the reaction conditions, ethylene glycol reacts with the acetate ions as follows:


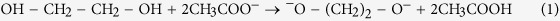


Thus, the EG molecules loses protons and coordinate with iron ions generating alkoxides.


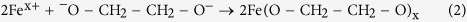


At elevated temperatures and under hydrothermal conditions, these alkoxides decomposes to generate Fe_3_O_4_ nanocrystals. Subsequently, these Fe_3_O_4_ nanocrystals grow larger and gets assembled together generating spherical porous structures. PVP was used as a capping agent which provides an effective coverage of the secondary nanostructures resulting in uniform spherical porous Fe_3_O_4_ secondary nanostructures.

The crystalline structure and the crystallite size of the so-prepared MFSNs are studied by powder X-ray diffraction analysis (Supplementary Fig. 1). The diffraction peaks at 2θ = 30.1, 35.8, 43.1, 53.8, 57.3, 63.0 corresponding to the (220), (311), (400), (422), (511) and (440) reflection planes are in well agreement with the cubic phase of Fe_3_O_4_ with a fcc crystal structure (JCPDS file no. 89-0951). The broadened diffraction peak profile indicates the presence of smaller crystallites. Based on the single line analysis^22^, which employs a pseudo-Voigt profile shape function, the crystallite size is calculated to be 7.73 nm with a lattice strain of 6.34 × 10^−2^. The SEM micrograph (Supplementary Fig. 2) shows that the synthesized Fe_3_O_4_ structures are uniform and spherical in shape with low poly-dispersity and an average particle size of around 500 nm. The Energy-dispersive X-ray (EDX) spectrum of the Fe_3_O_4_ structures confirms the presence of Fe and O only.

**Figure 1:**
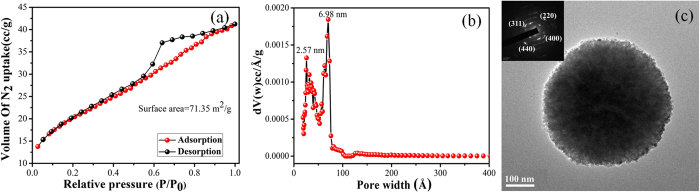
(**a**) Nitrogen adsorption-desorption isotherm, (**b**) Pore-size distribution curve and (**c**) TEM image and SAED pattern (inset) of MFSNs.

Specific surface area and pore size distribution of MFSNs are determined by measuring the adsorption and desorption isotherms of N_2_ (Fig. 1a,b). Prior to the N_2_ adsorption studies, the sample is degassed in vacuum at 250 °C for a few hours. It is evident from Fig. 1a that the N_2_ adsorption-desorption curve is a typical Type IV adsorption isotherm which displays a hysteresis loop of type H_2_. Such type of hysteresis is displayed in case of systems having complex interconnectivity of pores which can be understood from the sharp step in the desorption isotherm. The distribution of pore sizes and the pore shape is not well-defined or irregular in such systems. Figure 1b displays the corresponding pore size distribution (PSD) curve which is calculated from the adsorption branch of the isotherm using the Barrett-Joyner-Halenda (BJH) method. Figure 1b shows that the size of the pores ranges from 2 to 7 nm which is a representative of mesoporous structures. The Brunauer-Emmett-Teller (BET) specific surface area of MFSNs, calculated from the nitrogen adsorption analysis is found to be 71 m^2^/g.

Transmission electron microscopy (TEM) study of MFSNs (Fig. 1c) further confirmed the structure and morphology of the MFSNs. The representative high resolution transmission (HRTEM) image established the presence of highly mesoporous network in the MFSNs. The selected area electron diffraction (SAED) pattern of MFSNs is depicted in the inset of Fig. 1c, where polycrystalline nature of the microstructures can be confirmed. Here, the SAED pattern analysis confirms presence of (220), (311), (440), (400) reflection planes which are in well agreement to that obtained from the XRD pattern.

Magnetic properties of the MFSNs are evaluated by studying the field dependent magnetization behavior at room temperature and at low temperature. From the M-H curves (Supplementary Fig. 3), it is observed that the saturation magnetization value at room temperature (i.e. 300 K) is 77.68 emu/g and at 5 K it increases to 83.89 emu/g. It is because of the fact that the magnitude of thermal vibrations of atom increases with increasing temperature of the solid. This increased thermal motion of the atoms results in randomizing the direction of the aligned magnetic moment which results in decreasing the values of saturation magnetization, retentivity and coercivity. Hence, it is worthwhile to mention that at low temperatures, the thermal vibration is minimum which enhances the magnetization values. The effect of temperature over magnetization is studied (Supplementary Fig. 4) using ZFC (zero field cooling) and FC (field cooling) protocols at a field of 500 Oe. For ZFC measurements, the sample is allowed to cool in zero magnetic field from 300 K to 5 K and subsequently magnetization is measured while warming the sample again to 300 K with a probe field of 500 Oe. While in case of FC measurements, sample is cooled from 300 K to 5 K in presence of a magnetic field and subsequently magnetization measurements are recorded while increasing the temperature to 300 K. From the graph, it can be seen that the ZFC curve of MFSNs do not collapse anywhere into the FC curve. The observed pattern in the ZFC-FC curve implies that the blocking temperature of secondary nanostructures is well above the room temperature. It is because a large fraction of particles at room temperature are still in the blocked state which results in a broad distribution of the anisotropy energy barriers for the re-orientation of magnetic moments. This may be due to the strong interactions occurring between the nanoparticles within the MFSNs^23^.

### Heavy metal adsorption studies

**Figure 2:**
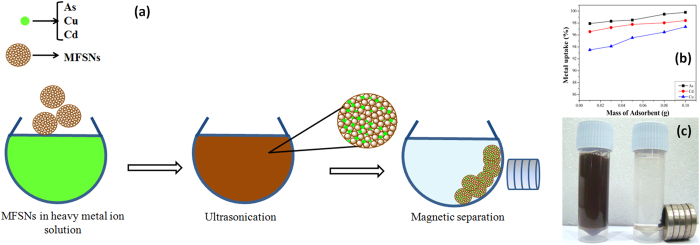
(**a**) Schematic showing scavenging of heavy metals, (**b**) Uptake of As, Cu, Cd as a function of the mass of adsorbent and (**c**) Photograph showing easy magnetic separation of As using MFSNs using low magnetic field.

The heavy metal removal efficiency of the MFSNs is studied with varying amount of adsorbent dosage: 0.01, 0.03, 0.05, 0.08 and 0.1 g at 80 min contact time with 0.5 ppm of heavy metal solution. From the Fig. 2, it is observed that with increase in the amount of adsorbent from 0.01 g to 0.1 g there is a sharp increase in percentage removal of the heavy metal (As, Cu and Cd) ions. The maximum percentage removal of As, Cu and Cd is found to be 99.8%, 97.3% and 98.4% respectively at an adsorbent dose of 0.1 g. It is evident that the removal of heavy metals increases rapidly with the increase in the adsorbent dosage which can be attributed to the availability of increased adsorption sites. The porous nature of the adsorbent aids to the surface area which in turn increases the number of accessible adsorption sites for the adsorbate to get attach.

Once the heavy metal species get adsorbed to the MFSNs, they can be easily removed from aqueous solution using a low magnetic field. The inset in Fig. 2 depicts the process of the easy manipulation of the heavy metal sorbed adsorbate using button magnet. The initial rust-colored solution contains MFSNs homogeneously dispersed in heavy metal solution. Upon agitation for a short contact time of 20 mins, the heavy metal ions got adsorbed to the surface of the adsorbate. It is seen that on application of the magnetic field, the solution becomes clear within a very short span of time and the particles get accumulated at the bottom of the vial where the magnetic field is applied.

It is observed from BET studies that the pore sizes of MFSNs range from 2 to 7 nm. It is worthwhile to mention that the heavy metal species viz. As, Cu and Cd have an atomic diameter of sizes 0.27, 0.25 and 0.3 nm respectively. So, the highly efficient uptake of the heavy metal species by the porous adsorbate can be corroborated to the fact that fine dimension heavy metal ion species are easily sequestered in the pores of the MFSNs.

### Effect of initial concentration of the heavy metals

**Figure 3:**
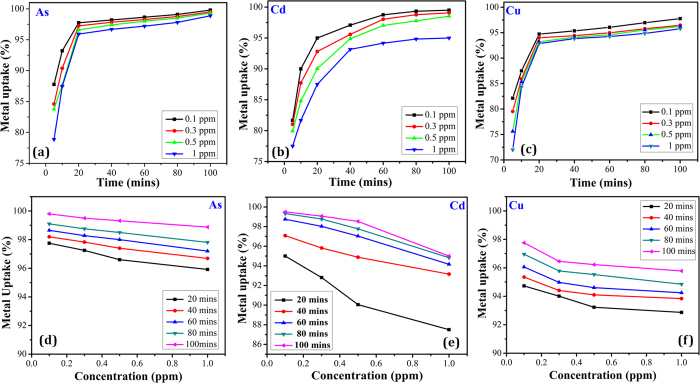
Initial concentration variation and contact time variation curves of (**a,d**) As (**b,e**) Cu and (**c,f**) Cd respectively.

In the present study, the initial concentration of the heavy metal ions chosen for the adsorption studies are almost in equivalence to the permissible limits prescribed by World Health Organization (WHO). Studies on the effect of initial concentration of the heavy metal species were carried out by varying it from 0.1 ppm to 1 ppm with an adsorbent dosage of 0.05 g at pH 7. It is seen from Fig. 3a–c that the percentage removal decreases with the increase in initial heavy metal concentration. The adsorption efficiency is higher in case of 0.1 ppm initial concentration of each of the three heavy metals, after which it decreases gradually. The maximum removal percentage of As, Cu and Cd at 0.1 ppm concentration is 97.8%, 94.8% and 96.8% respectively. Hence, it is seen that the remaining concentration of arsenic, copper and cadmium after adsorption in the test solution is 0.0022 ppm, 0.0052 ppm and 0.0032 ppm respectively, which are well below the WHO prescribed limits for As (0.01 ppm), Cu (1.3 ppm) and Cd (0.005 ppm).

It is apparent that at lower metal ion concentrations, sufficient adsorption sites are available for adsorption of the heavy metal ions. However, with increase in the concentration of the metal ions the availability of free adsorption sites becomes scarce which affects the rate of adsorption. Hence, it is seen that the percent removal of heavy metals directly depends on the initial metal ions concentration and decreases with increase in metal ion concentration.

### Effect of contact time

The correlation between the adsorption of As, Cu and Cd metal ions and contact time is investigated to study the rate of heavy metal adsorption. The percent removal is studied with 0.05 g of adsorbent and varying initial heavy metal ion concentration ranging from 0.1 to 1 ppm at different contact time period of 5, 10, 20, 40, 60, 80 and 100 min. From Fig. 3d–f, it is observed that there is a sharp increase in percentage removal of As, Cu and Cd at 20 min of contact time, followed by gradual increase with almost absolute removal (~99%) at 100 min. The rapid adsorption at the initial contact time may be due to the counter attraction between the negatively charged surface of the adsorbent and the cationic heavy metal ions. With the advancement of time, a repulsive force comes into play between the charged metal ions which slows down the adsorption process.

### Adsorption Kinetics

The study of adsorption kinetics describes the rate of adsorption and evidently this rate controls the dwelling time of the adsorbate uptake at the solid-solution interface^23^. Kinetic study of As, Cu and Cd adsorption by the MFSNs are analyzed using pseudo first-order and pseudo second-order models. To analyze the adsorption kinetics of the heavy metals, pseudo first order and pseudo-second order kinetics has been carried out from the data obtained from experimental results.

**Figure 4:**
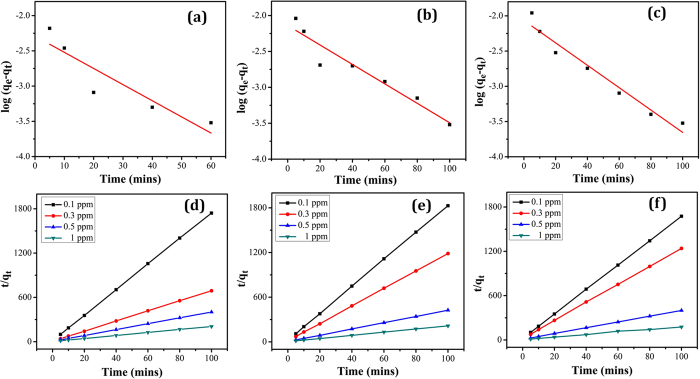
Pseudo-first order and Pseudo-second order kinetic adsorption model plots for the adsorption of (**a,d**) As, (**b,e**) Cu and (**c,f**) Cd respectively.

Pseudo first-order kinetics is described by the following equation


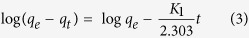


where, q_e_ and q_t_ are the amounts (mg/g) of adsorbate at equilibrium and at time t (s), respectively, and K_1_ is the rate constant of pseudo first- order adsorption. The plot of log (q_e_ − q_t_) versus t gives a straight line from which K_1_ and q_e_ can be determined from the slope and intercept respectively. Figure 4(a–c) represents the log (q_e_ − q_t_) versus t plot for As, Cu and Cd adsorption. The K_1_ and q_e_ values for each heavy metal system are calculated from these pseudo first-order kinetic plots (Supplementary Table 1). It is known that if the calculated q_e_ does not equate to the experimental q_e_, then the reaction is not likely to be a first-order reaction^24^. From the experimental datas, it can be observed that the calculated q_e_ values are too low compared to experimental q_e_ values. Thus, it can be concluded that adsorption of As, Cu and Cd by the MFSNs does not follow pseudo first-order kinetics.

The linearized form of the pseudo second-order kinetics is shown in equation 4,


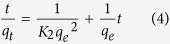


where K_2_ is the second order rate constant of adsorption. As shown in Fig. 4(d–f), the t/q_t_ versus t plots for As, Cu and Cd adsorption yields a linear relationship from which K_2_ and q_e_ are determined. The calculated q_e_ values fit well with the experimental q_e_ values and the correlation coefficient R^2^ is high (~0.99), which indicates that the adsorption of As, Cu and Cd on MFSNs follows pseudo second-order kinetics. Thus, it can be concluded that the heavy metal ions are adsorbed on to the surface of the adsorbent through valence forces that involves the sharing or exchange of electrons between the sorbent and sorbate. From the second-order kinetic analysis, a higher K_2_ value is obtained for As in comparison to Cu and Cd. The higher K_2_ values generally indicates the faster removal of As by the adsorbent in comparison to Cu and Cd.

It is seen that the R^2^ values in case of pseudo first-order kinetics for all the heavy metal species is less as compared to the R^2^ values for pseudo second-order reaction (Supplementary Table 1).

### Adsorption Isotherm

The adsorption isotherm model identifies the nature of interaction between the adsorbent and the adsorbate when the adsorption process reaches equilibrium. The experimental data from the study of As, Cu and Cd adsorption by MFSNs are analyzed using Langmuir, Freundlich and Temkin isotherm equations.

Langmuir isotherm is valid for monolayer adsorption and is based on the assumption that adsorption sites have equal affinity for the molecules of the adsorbate^25^. According to this isotherm, maximum adsorption takes place when a saturated monolayer of solute molecules is present on the adsorbent surface, the energy of adsorption is constant and there is no migration of adsorbate molecules in the surface plane. Mathematically, it is defined as


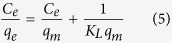


where q_m_ and K_L_ are Langmuir constants representing the maximum adsorption capacity of adsorbents (mg/g) and the energy of adsorption, respectively. The values of q_m_ and K_L_ can be calculated from the slope and intercept of the linear plots C_e_/q_e_ versus C_e_.

Freundlich isotherm is based on multilayer adsorption over heterogeneous surfaces highlighting the fact that the adsorption sites are distributed exponentially with respect to the heat of adsorption^24^. The linearized form of the Freundlich equation is depicted by mathematical expression as


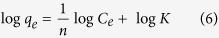


where q_e_ is the amount of adsorbate, C_e_ (mg/l) is the equilibrium solute concentration in solution, and K_F_ and n are Freundlich constants related to adsorption capacity and adsorption intensity, respectively. The values of K_F_ and n can be calculated from the slope and intercept of the plot of log q_e_ against log C_e_.

Temkin isotherm assumes the fact that the effects of the heat of adsorption decreases linearly with coverage of the adsorbate and adsorbent interactions^26^. The linearized form of the Temkin equation can be expressed as


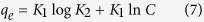


where, K_1_ and K_2_ are Temkin constants, whose values can be calculated from the plots between q_e_ and ln C_e_. The constants K_1_ and K_2_ represent heat of adsorption and equilibrium binding constant respectively.

The adsorption capacity of all the three heavy metal species on MFSNs is calculated from the Langmuir adsorption isotherm. It is found that the MFSNs exhibited a much higher adsorption capacity for As (0.870 mmol/g) than Cu (0.4723 mmol/g) and Cd (0.691 mmol/g). The higher absorption capacity of MFSN for As can be understood from the fact that iron oxide based adsorbents have a strong affinity towards As. The value of the correlation coefficient (R^2^) for Langmuir equation for adsorption of As(V) and Cu(II)is much higher (0.994 and 0.991) than that for Freundlich (0.901 and 0.828) and Temkin equation (0.881 and 0.899) respectively (Supplementary Table 2). Thus it can be concluded that the adsorption of As(V) and Cu(II) fits well with the Langmuir isotherm, indicating the binding energy over the entire surface of the adsorbate is uniform and there is no significant interaction among the adsorbed species. The adsorption data interprets that the materials are adsorbed forming a monolayer on a homogeneous surface. This phenomenon also indicates that chemisorption is the principal mechanism involved in the sorption process. For As(V) as well as Cu(II), there exists an electrostatic attraction between negatively charged Fe_3_O_4_ samples surface and positively charged As(V) and Cu(II) species and the adsorbed heavy metal species have a repulsive effect on As(V) and Cu(II) species present in the solution.

The Langmuir, Freundlich and Temkin constants for the adsorption of Cd(II) were calculated from the linear plot and the correlation coefficients (R^2^) (Supplementary Table 2). From the adsorption data, it is observed that the correlation coefficient (R^2^) for Cd(II) adsorption has much higher value for Freundlich equation (0.998) than the Langmuir (0.959) or Temkin (0.897) equation. Moreover, the value of n obtained (1.85) represents a physical process which implies that mesoporous MFSNs has a high affinity for Cd(II) ions in solution. The higher K_F_ value obtained from the Freundlich isotherm for Cd adsorption indicates the high adsorption capacities. Thus, from these two facts it can be interpreted that Cd(II) adsorption fits well with Freundlich isotherm and the process follows multilayer adsorption on a surface of the adsorbate.

## Discussions

In summary, unique characteristics of the MFSNs such as large surface area, enhanced adsorption sites in the form of porous microsphere and inherent magnetic properties makes it a promising candidate for heavy metal remediation. In the present work, the influence of the experimental conditions over the adsorption phenomenon has been evaluated. Batch experiment studies elucidated that the adsorption process is very fast and within a very short contact time the adsorbent showed high removal efficiency of all the heavy metal species. Fitting equilibrium data to the Langmuir, Freundlich and Temkin isotherms revealed that the As(V) and Cu(II) adsorption study are in good agreement with the Langmuir isotherm whereas Cd(II) adsorption fits well with the Freundlich isotherm. Hence, it can be inferred that the MFSNs has considerable potential as a versatile heavy metal adsorbent for effective remediation within a very short contact time. Moreover, MFSNs are amenable to easy filtration, forbidding draining out of the metal adsorbed grains through the filters during filtration process. The regenerative study of the adsorbent is a future prospect of this work.

## Materials

### Chemicals

Ferrous chloride (FeCl_3_), sodium acetate (NaOAc), ethylene glycol, ethanol are procured from Merck Specialities Pvt. Ltd. Polyvinylpyrrolidone (PVP) is procured from Sigma-Aldrich. All the chemicals are of analytical grade and used as received without further purification. The de-ionised water used in this study is obtained from a Milli-Q water system.

### Synthesis procedure for the development of MFSNs

The development of MFSNs involves a modified template free hydrothermal procedure^20^. In a typical procedure, 1.5 g FeCl_3,_ 1 g PVP and 2 g NaAc are added to 30 ml of ethylene glycol and subjected to vigorous stirring for 2 hours to get dissolved completely. Then, the mixture is transferred to a Teflon- lined stainless steel autoclave and heated at a temperature of 200 °C for 8 hours. After this reaction duration, the autoclave is allowed to cool naturally to room temperature. The resulting black solution is then centrifuged to separate the secondary nanostructures. Subsequently, the precipitate obtained is washed with ethanol followed by distilled water for a few times to remove the impurities and finally allowed to dry in vacuum at 60 °C for 24 hours in order to obtain fine black powder.

### Preparation of heavy metal stock solution

The stock solutions of arsenic, copper and cadmium are prepared in de-ionized water from standard heavy metal solutions. The pH of the stock solutions is adjusted to neutral using 0.1% TMAOH or HCl.

### Characterization

The phase analysis of the prepared samples is performed using Rigaku CD 100 41 XRD unit with copper target having a wavelength 1.54 Å. The microstructure is studied with the aid of scanning electron microscopy (JEOL JSM Model 6390 LV) and transmission electron microscopy (JEOL JEM-2100, 200 kv. The Brunauer-Emmett-Teller (BET) surface area and the pore size distribution plots are calculated by applying the linear part of the BET plot and the Barrett-Joyner-Halenda (BJH) model which is performed using Autosorb 1C, Quanta Chrome, USA instrument operated at 77 K. The magnetic properties of the so prepared samples are investigated using Quantum Design 9T PPMS with an applied field of 500 Oe at room temperature and low temperature. The concentrations of the heavy metal in aqueous solutions are measured using a Thermo Scientific Atomic absorption spectrometer (AAS-ICE 3500).

### Batch adsorption experiment

Batch adsorption studies are conducted to observe the effect of adsorbent dosage, initial heavy metal concentration and contact time. Solutions containing different concentrations (0.1, 0.3, 0.5 and 1 ppm) of As, Cu and Cd are prepared and adjusted to pH 7 using TMAOH/HCl. It is known that the surface charge of the adsorbent plays an important role in the adsorption process. Therefore, before carrying out the adsorption measurements the surface charge of the adsorbent in a pH range of 2 to 12 (in all the three test heavy metal solutions of strength 0.1 ppm)has been studied and it is found that the adsorbent is most stable in the pH range of 6–7. As a result all the adsorption studies are carried out at neutral pH. A pre-determined weight of MFSNs (i.e. 0.05 g adsorbent) is added to 25 ml of each heavy metal (As, Cu and Cd) solution of known concentration at room temperature. The heavy metal solutions are agitated by ultrasonication to achieve complete equilibrium. The adsorbent is then separated magnetically at regular interval and the concentration of the heavy metals left in the aqueous solution is determined using AAS. For evaluating the effect of adsorbent dosage, another batch of adsorption study is carried out with 25 ml of the heavy metal (i.e. As(V), Cu(II) and Cd(II)) solution of known concentration (i.e. 0.5 ppm) for a fixed period of contact time (i.e. 80 minutes) with different weight of adsorbent (0.01, 0.03, 0.05, 0.08 and 0.1 g) respectively. After specified time, the two phases are magnetically separated and the supernatant is then analyzed for the residual heavy metals concentration using AAS. Heavy metal solution of known concentration (0.5 ppm) containing a fixed amount of adsorbent dosage (0.05 gm) are studied for adsorption at different contact time varying from 5 to 100 mins. The results of these studies are used to obtain the optimum conditions for maximum heavy metals removal from aqueous solution. The percentage of heavy metal removal is calculated according to the following equation


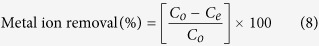


where, C_o_ is the initial metal ion concentration of test solution (mg/l), C_e_ is the final equilibrium concentration of test solution(mg/l). The equilibrium adsorption capacity (Q_e_) (mg/g) for arsenic, copper and cadmium is calculated according to the following equation


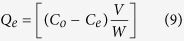


where, V is the volume (ml) of heavy metal aqueous solution and W is the weight (mg) of adsorbents used in the experiment.

## Additional Information

**How to cite this article**: Bhattacharya, K. *et al.* Mesoporous magnetic secondary nanostructures as versatile adsorbent for efficient scavenging of heavy metals. *Sci. Rep.* **5**, 17072; doi: 10.1038/srep17072 (2015).

## Supplementary Material

Supplementary Information
