# Supplementary material for: Mesoporous magnetic secondary nanostructures as versatile adsorbent for efficient scavenging of heavy metals
Source: Sci Rep. 2015 Nov 25;5:17072. doi: 10.1038/srep17072 (PMC4658508; doi:10.1038/srep17072)
Supplement: Supplementary Information [file srep17072-s1.doc]

**Mesoporous magnetic secondary nanostructures as versatile adsorbent for efficient scavenging of heavy metals**

Kakoli Bhattacharya^1^, Devaborniny Parasar^1^, Bholanath Mondal^2^ and Pritam Deb^1^*

^1^ Department of Physics, Tezpur University (Central University), Tezpur-784028, India

^2^ Department of Central Scientific Services, Indian Association for the Cultivation of Science, Jadavpur, Kolkata 700032, India

***Corresponding Author Email:** *pdeb@tezu.ernet.in*


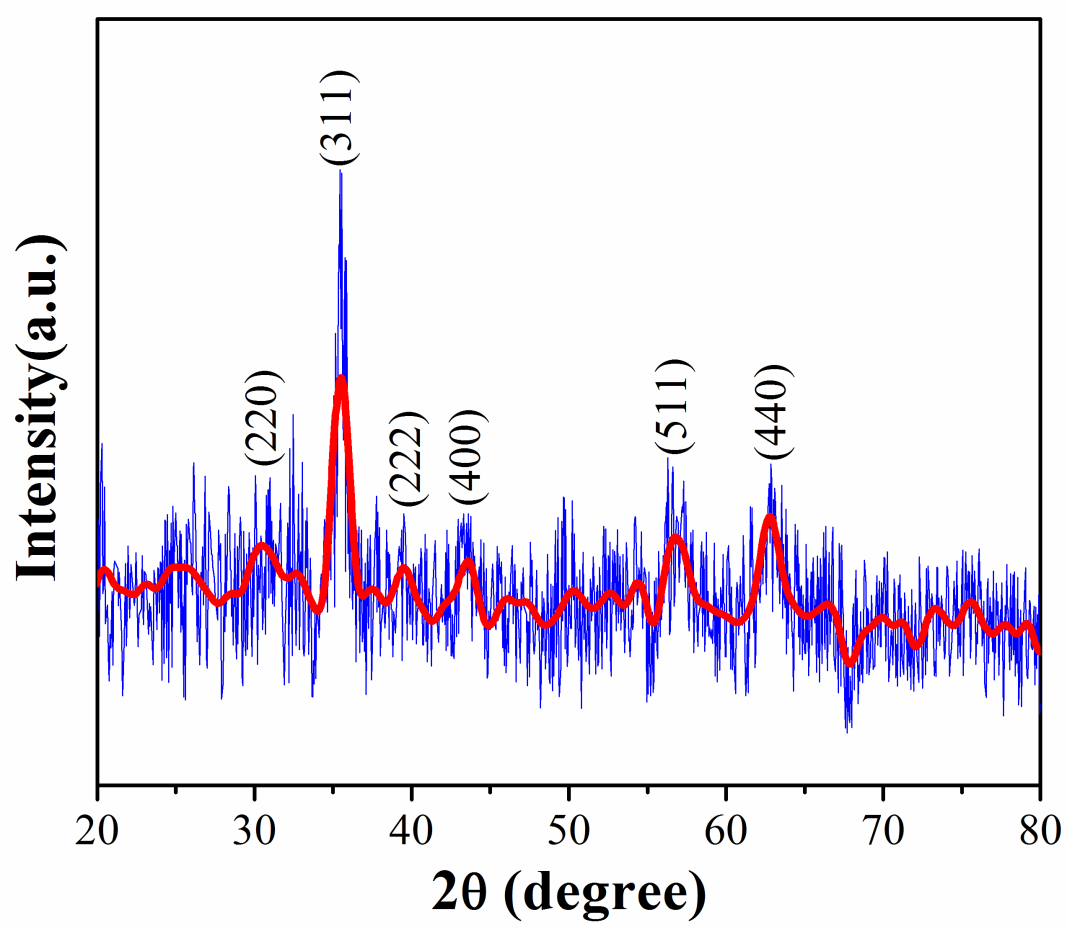


**Supplementary Figure 1:** Powder X-ray diffraction patterns of mesoporous Fe_3_O_4_ secondary nanostructures (MFSNs)


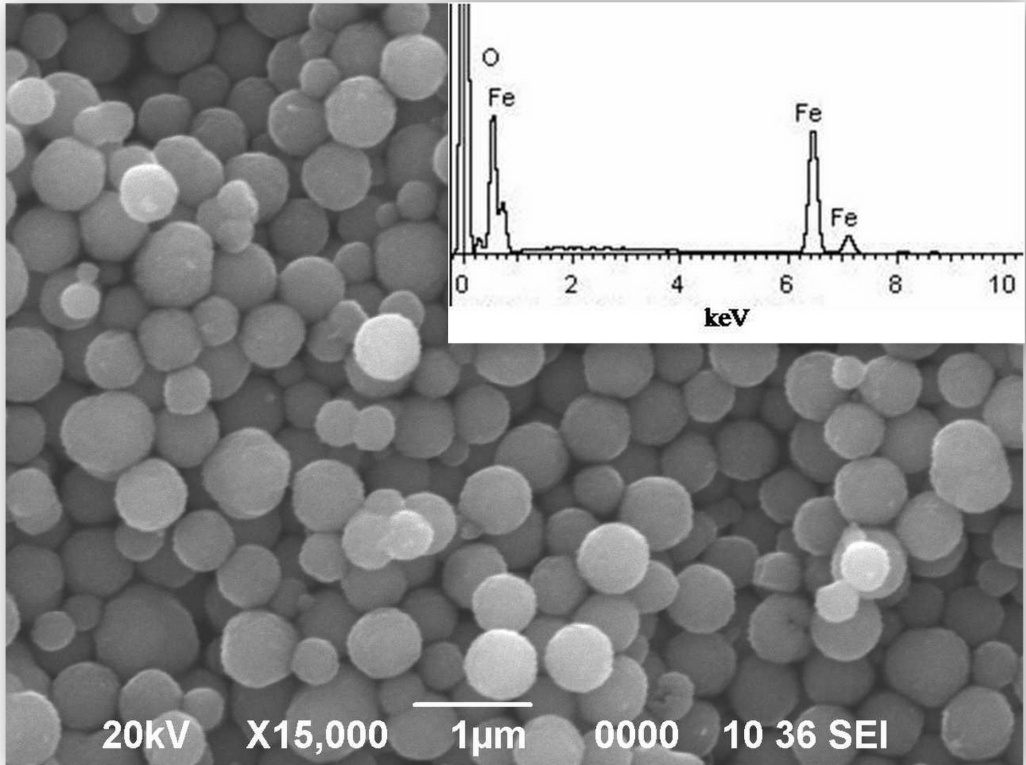


**Supplementary Figure 2:** Scanning electron micrograph (SEM) of MFSNs with the inset showing the energy-dispersive X-ray (EDX) spectrum


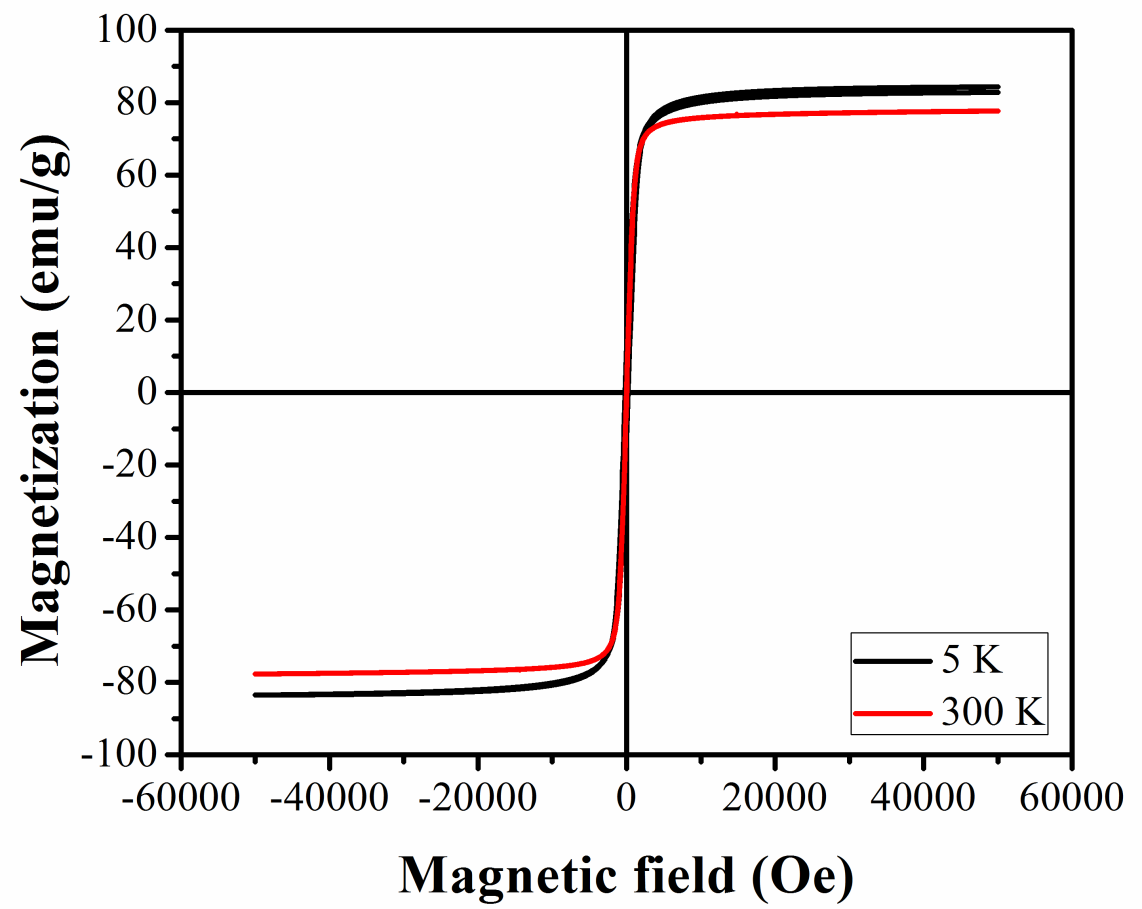


**Supplementary Figure 3:** Field dependent magnetization studies of MFSNs at 5K and 300K


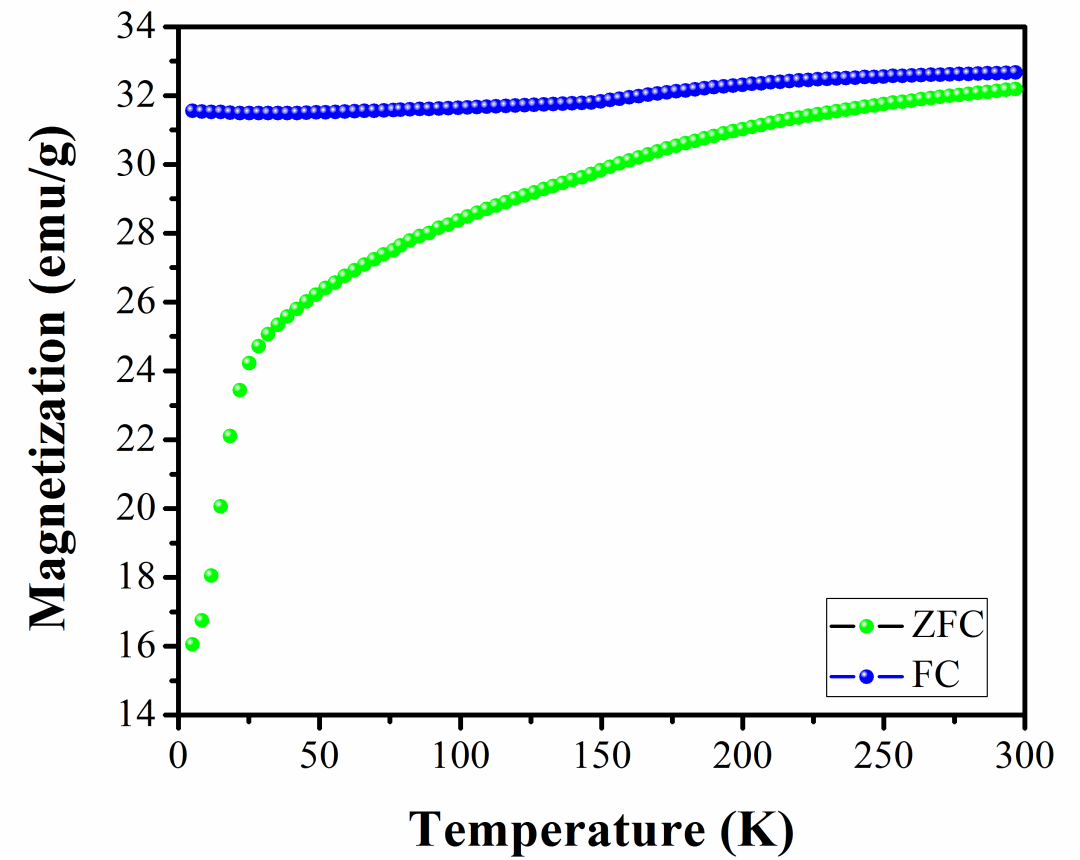


**Supplementary Figure 4:** Temperature dependent magnetization studies of MFSNs at a field of 500 Oe

**Supplementary Table 1: Kinetic Parameters for the adsorption of As, Cu and Cd metal ions**

| Isotherms |  | Arsenic | Copper | Cadmium |
| --- | --- | --- | --- | --- |
| Pseudo-first order | K_1_  q_e_  R^2^ | 0.052  0.005  0.793 | 0.029  0.007  0.911 | 139.253  7.177  0.952 |
| Pseudo-second order | K_2_  q_e_  R^2^ | 0.092  0.490  0.999 | 0.0001  0.055  0.999 | 0.0001  0.060  0.999 |

**Supplementary Table 2: Equilibrium Adsorption Isotherm Fitting Parameters for As, Cu and Cd**

| Isotherms |  | Arsenic | Copper | Cadmium |
| --- | --- | --- | --- | --- |
| Langmuir | q_m_(mmol/g)  K_L_  R^2^ | 0.870  60.496  0.994 | 0.4723  357.142  0.991 | 0.691  70.422  0.959 |
| Freundlich | 1/n  K_F_  R^2^ | 0.373  2.363  0.901 | 0.578  2.220  0.828 | 0.538  2.588  0.998 |
| Temkin | K_1_  K_2_  R^2^ | 0.136  1104.078  0.881 | 0.148  313.328  0.899 | 0.118  1318.256  0.897 |
